# Non-clinical isolates as potential reservoirs of antibiotic resistance in Port Harcourt, Nigeria

**DOI:** 10.11604/pamj.2018.30.167.14261

**Published:** 2018-06-25

**Authors:** Kome Otokunefor, Paul Agbude, Tosanwumi Vincent Otokunefor

**Affiliations:** 1 Department of Microbiology, Faculty of Science, University of Port Harcourt, Rivers State, Nigeria

**Keywords:** MDR, reservoir, Nigeria, Escherichia Coli, Pseudomonas aeruginosa

## Abstract

**Introduction:**

Multidrug resistance (MDR) is a growing problem worldwide. This type resistance often arises due to the sequential acquisition of drug resistance determinants and subsequent clonal spread. It is therefore important to determine possible reservoirs of these MDR gene to help set out control strategies. This study was aimed at analysing susceptibility patterns of various non-clinical Gram negative bacterial strains to determine their potential as reservoirs of MDR.

**Methods:**

Thirty-five non-clinical Gram negative bacteria were identified and susceptibility profile determined using standard methodologies.

**Results:**

Findings showed a preponderance of *Pseudomonas aeruginosa* and *Escherichia Coli*. Resistance rates of above 80% were noted in 50% of antibiotics, though none of the isolates were resistant to Ofloxacin. Majority of isolates (68.6%) had a multiple antibiotic resistance (MAR) index greater than 0.5, but only 20% of *Escherichia Eoli*. were found in this category. A high level of MDR was noted in this study (71.4%), but again only 20% of these were *Escherichia Coli*.

**Conclusion:**

Gram negative bacteria are the most common group of bacteria frequently encountered in clinical microbiology. In more recent years, infections with these organisms have been further complicated by the phenomenon of drug resistance. Non-clinical isolates have been postulated as possible reservoirs. Findings from this study of widespread multidrug resistance support this idea. This study however highlights the lack of MDR in *Escherichia Coli*, which is promising. More extensive studies will need to be carried out to properly assess the role of non-clinical isolates as reservoirs of MDR determinants.

## Introduction

The problem of multidrug antibiotic resistance is an ever growing one, with increasing reports on ‘superbugs’ made worldwide [1]. Multidrug resistance (MDR) has been defined as resistance or non-susceptibility of an organism to at least one drug from 3 or more defined classes of antibiotics [2]. This phenomenon is commonly thought to result from the sequential acquisition of drug resistance determinants and clonal dissemination following an index case of point mutation rather than from widespread random point mutations. Notable examples of MDR bacteria have been described and include *Carbapenem-resistant Enterobacteriaceae* (CRE), Vancomycin-resistant *Enterococcus* (VRE) and Methicillin-resistant *Staphylococcus aureus* (MRSA) [3, 4]. Carbapenem-resistant *Klebsiella pneumoniae* which is a notable example of CRE, is characterised by the presence of mobile genetic elements (MGEs) carrying genes encoding resistance to beta lactams, Aminoglycosides, Macrolides and Quinolones [5]. A recent report on a pandrug resistant isolate with resistance to Colistin even noted that this resistance was caused by the functional inactivation of a gene due to the insertion of a MGE that encodes resistance to Carbapenem [3]. Similarly, strains of VRE were shown to be resistant to a number of other antibiotics (Tetracycline, Erythromycin, Streptomycin and Gentamicin). The genes for these were carried on two conjugative transposons [6]. A similar trend has also been reported for MRSA [7]. High level MDR has been reported in several non-clinical isolates, with some studies suggesting that these isolates could act as sources of antimicrobial resistance (AMR) determinants to clinical strains [8-10]. This level of resistance, sometimes to the last line drugs effective against a group of microorganisms, has raised fears of a return to the pre-antibiotic era and the associated high mortality rates. While the development of resistance definitely appears to be driven by the selective pressure of antibiotic used in clinical settings, there is still a significant role for traffic of MGE even across species boundaries outside of clinical settings. In order to curb the development of drug resistance, it has become essential to properly assess possible reservoirs of MDR determinants. Over the years numerous reservoirs of drug resistance have been reported elsewhere [8, 11]. Few studies have been carried out in Nigeria focused on assessing the role of non-clinical isolates to serve as reservoirs of multidrug resistance. This study therefore set out to analyse the susceptibility patterns of various non-clinical Gram negative bacteria in order to determine the potential of these bacteria as reservoirs of MDR.

## Methods

**Bacterial isolation and characterisation:** Thirty-five Gram negative bacteria were isolated from various non-clinical sources affected by human interaction. These sources included, surface water (7), slaughter houses (9), cloak rooms of male and female hostels (19). These organisms were characterised using standard biochemical tests to determine their identities [12, 13].

**Antibiotic resistance testing:** Resistance profile of each organism was determined using the Kirby Bauer disc diffusion technique [14]. This method involves first plating out a 0.5 McFarland concentration of inoculum unto the surface of a sterile Mueller Hinton Agar plate, followed by the application of a standard Gram negative multidisc (Ceftazidime (30 µg), Cefuroxime (30 µg), Gentamicin (10 µg), Cefixime (5 µg), Ofloxacin (5 µg), Augmentin (30 µg), Nitrofurantoin (300 µg) and Ciprofloxacin (5 µg)). Resistance profile was then generated for each organism from a standard [15] based on the zones of inhibition observed following a 24 hour incubation at 37οC.

**Determination of MAR index and multidrug resistance:** The MAR index points at the level of resistance exhibited by each organism. This was calculated as a/b where "a" is the total number of antibiotic to which the organism was resistant and "b" is the total number of antibiotics against which the organisms were tested [16]. Multidrug resistance was then determined by ascertaining the drug class of each test antibiotic and noting those organisms with resistance to three or more classes.

## Results

**Bacterial identification:** Of the 35 Gram negative organisms isolated, *Pseudomonas aeruginosa* and *Escherichia Coli* were the two predominant organisms detected in this study with frequencies of 31.4% and 28.8% respectively, making up 60.2% of the total isolates. Other organisms identified include *Klebsiella pneumoniae* (11.4%), *Aeromonas sp* (11.4%), *Serratia marcescens* (11.4%) and *Salmonella sp* (5.7%).

**Figure 1:**
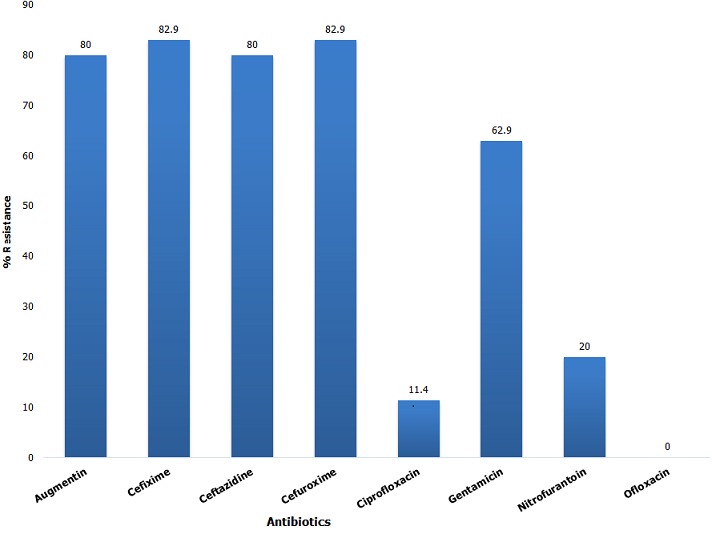
Comparative resistance rates to individual antibiotics

**Figure 2:**
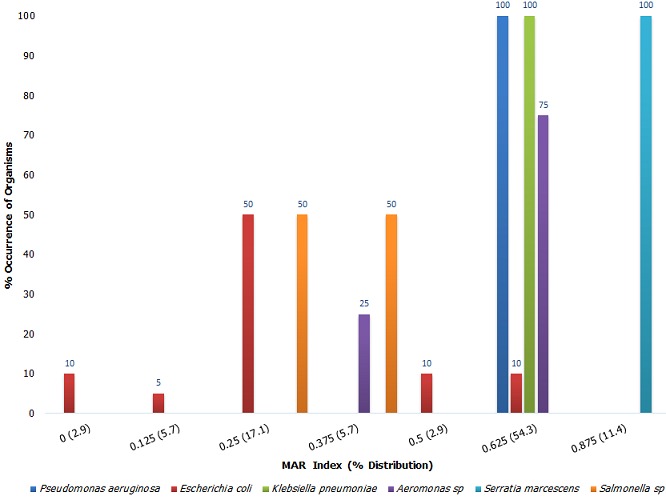
MAR index of test isolates

**Antibiotic susceptibility profile:** An analysis of the susceptibility profile of the test organisms revealed a high degree of resistance to antibacterial agents (Figure 1). Of the 8 antibiotics tested, resistance rates of above 80% were noted in 50% of antibiotics. The lowest level of resistance was noted with Ofloxacin (0%). In total, 13 antibiotic resistance profiles were observed (Table 1). Majority (18/35, 51.4%) of the isolates were resistant to Augmentin, Ceftazidime, Cefuroxime, Cefixime and Gentamicin. Total susceptibility to all test antibiotics was observed in only one case and resistance to all test antibiotics was not noted in this study. Majority of test isolates (68.6%) had a MAR index above 0.5 (Figure 2). An analysis of the data however, shows that the higher MAR index values were mainly found with *P. aeruginosa, K. pneumoniae, Aeromonas sp*and *Salmonella sp.* Only 20% of the *Escherichia Coli* isolates had a MAR index of 0.5 and above. An assessment of MDR based on the 2011 Magiorakos publication [2] showed that most of the organisms (25/35, 71.4%) were MDR. MDR was however observed in only 2 (20%) of the *Escherichia Coli* isolates (Table 2), majority of the *Escherichia Coli* isolates (80%) were rather non-MDR.

**Table 1:** Antibiotic resistance profile of bacterial isolates

Organism ID	Resistance Profile	Number of Organisms
1	-	1
2	CRX	2
3	AUG-CRX	1
4	AUG-NIT	1
5	CAZ-CXM	3
6	CXM-CAZ	1
7	AUG-CRX-CXM	1
8	AUG-CRX-NIT	1
9	AUG-CAZ-CRX-CXM	1
10	AUG-CAZ-CRX-CXM-GEN	18
11	AUG-CAZ-CRX-CXM-NIT	1
12	AUG-CAZ-CPR-CRX-CXM-GEN-NIT	4

AUG: Augmentin; CAZ: Ceftazidime; CPR: Ciprofloxacin; CRX: Cefuroxime; CXM: Cefixime; GEN: Gentamicin; NIT: Nitrofurantoin.

**Table 2:** MDR in non-clinical isolates

Organism (n)	MDR Isolates	Antibiogram
*Pseudomonas aeruginosa* (11)	11	AUG-CAZ-CRX-CXM-GEN
*Escherichia coli* (10)	2	AUG-CAZ-CRX-CXM AUG-CAZ-CRX-CXM-NIT
*Klebsiella pneumoniae* (4)	4	AUG-CAZ-CRX-CXM-GEN
*Aeromonas* sp (4)	3	AUG-CAZ-CRX-CXM-GEN
*Serratia marcescens* (4)	4	AUG-CAZ-CPR-CRX-CXM-GEN-NIT
*Salmonella* sp (2)	1	AUG-CRX-NIT
**Total**	**25**	

AUG: Augmentin; CAZ: Ceftazidime; CPR: Ciprofloxacin; CRX: Cefuroxime; CXM: Cefixime; GEN: Gentamicin; NIT: Nitrofurantoin

## Discussion

Gram negative bacteria, particularly members of the enterobacteriaceae are the most common group of bacteria frequently encountered in the clinical microbiology laboratory. These organisms are notorious for the wide array of diseases that they cause, ranging from mild infections to more life threatening diseases. Furthermore, they are important nosocomial agents causing significant mortality in hospitalised patients with already immunocompromised systems. In more recent years, infections with these organisms have been further complicated by the phenomenon of drug resistance. Reports have been made of evolving resistance to Carbapenem, the last line drug effective against some Gram negative organisms. Several postulates have been made as to the sources of these resistance. One of these postulates is non-clinical isolates serving as reservoirs. In this study, similar to the situation in a clinical microbiology laboratory, the majority of non-clinical Gram negative organisms isolated were members of the enterobacteriaceae (20/35, 57.1%). This trend in prevalence of enterobacteriaceae detected was similar to that of a previous study with *Escherichia Coli* being the more common member of the family detected, followed by *K. pneumoniae* and then *Salmonella sp* [17]. An assessment of all test isolates revealed high rates of resistance to 5 out of the 8 antibiotics tested. These 5 antibiotics represent 3 broad drug classes, the aminoglycosides, penicillins and cephalosporins. Both penicillins and cephalosporins are traditional first-line therapy drug options for treating Gram negative bacteria in general and enterobacteriaceae in particular [18]. Several studies have reported high rates of resistance to these drugs worldwide, often resulting in therapy failure and a worse prognosis for the patients [17, 19]. This same trend is reflected in this current study where resistance rates of greater than 80% was noted for these two first line drugs. This high level resistance to first line drugs in non-clinical isolates should pose a major public health concern. Even more worrying is the high level resistance noted in this study against Gentamicin, an aminoglycoside. Though an older antibiotic, in more recent times there has been a tendency to use this drug to treat more serious Gram negative infections [20]. Therefore, high resistance rates in this class of drugs will further reduce available treatment options. One striking finding of this study was the 0% resistance to Ofloxacin reported. Despite some report of high level resistance to this drug [21, 22], similar low levels of resistance against Ofloxacin have been widely reported in Nigeria and elsewhere [23-25]. Ofloxacin a second generation quinolone antibiotic belongs to one of the most prescribed group of antibiotics worldwide. Like other quinolones, Ofloxacin acts on gyrase and topoisomerase IV enzymes, making them toxic with the ability to fragment bacterial chromosome [26]. Unlike the other quinonlones however, Ofloxacin is unique in that it lacks plasmid-borne resistance [27].

Despite these high rates of resistance to some drug classes, it is noteworthy that no isolate exhibited a 100% resistance to all antibiotics tested and may perhaps point at the fact that these isolates are still significantly less resistant than their clinical counterparts. Similarly, in this study, *Escherichia Coli* isolates were significantly less resistant than other isolates. Only 20% of the *Escherichia Coli* isolates had MAR index values of above 0.5 and were MDR. This is unlike previous reports from clinical studies which described MDR in up to 82.5% of the *Escherichia Coli* isolates [28]. This is encouraging as it indicates that these subset of non-clinical *Escherichia Coli* isolates are not major reservoirs of drug resistance. Additionally, the numerous susceptibility profiles associated with the *Escherichia Coli* isolates point at the circulation of multiple clones rather than the prevalence of a single clone. As a whole however, the rate of MDR detected in this study was quite high (71.4%) especially when compared to values ranging from 21.4% - 29.7% reported by previous studies on clinical isolates [29-31]. These rates were however similar to more recent studies reporting MDR rates ranging from 55.7% - 78%, also in clinical isolates [28, 32, 33]. Considering that apart from *Escherichia Coli* most of the other isolates had one or two antimicrobial sensitivity patterns, this could therefore have resulted from the circulation of a single clone of these isolates. Results from this study therefore show a high propensity of non-clinical isolates to serve as possible reservoirs of MDR genes. Further studies will need to be carried out to find out how widespread this trend is and also to determine possible driving determinants of this phenomenon in order to put adequate control measures in place. This study presents results on isolates obtained from a limited number of sampling sites. While these results provide baseline data, it cannot therefore be used to establish a trend. Further studies on a larger scale will need to be carried out to accomplish this. Additionally, this study reports on the ability of non-clinical isolates to act as potential reservoirs of antibiotic resistance based on their antibiotic resistance profile. Studies exploring the genetic mechanisms of these resistance will be necessary to contribute further to the understanding of this potential.

## Conclusion

Data on MDR in Nigeria is sparse. This study presents information on the possible role non-clinical Nigerian isolates play as reservoirs of MDR. Apart from the *Escherichia Coli* isolates, other Gram negative bacteria exhibited high rates of MDR, pointing at a possible role of these isolates as reservoirs of MDR. Further studies exploring the development and prevalence of these MDR non-clinical isolates are therefore necessary.

### What is known about this topic

- Multidrug resistance is increasing worldwide;
- Non-clinical isolates have already been reported as possible reservoirs of multidrug resistance.

### What this study adds

- This study presents new data on the current state of multidrug resistance in non-clinical isolates in Nigeria, adding to the current limited data on multidrug resistance in non-clinical isolates in Rivers State, Nigeria and highlighting the high rate of multidrug resistance, thereby indicating the need for more stringent control measures aimed at reducing the evolution of these organisms in non-clinical settings;
- This study shows an encouraging lack of multidrug resistance in non-clinical *Escherichia Coli* isolates;
- This study finds a lack of resistance to Ofloxacin among this group of non-clinical isolates, which would need to be explored further.

## Competing interests

The authors declare no competing interests.
